# Anthropometric criteria for best-identifying children at high risk of mortality: a pooled analysis of twelve cohorts

**DOI:** 10.1017/S136898002300023X

**Published:** 2023-04

**Authors:** Tanya Khara, Mark Myatt, Kate Sadler, Paluku Bahwere, James A Berkley, Robert E Black, Erin Boyd, Michel Garenne, Sheila Isanaka, Natasha Lelijveld, Christine McDonald, Andrew Mertens, Martha Mwangome, Kieran O’Brien, Heather Stobaugh, Sunita Taneja, Keith P West, André Briend

**Affiliations:** 1 Emergency Nutrition Network, ENN, 2nd Floor, Marlborough House, 69 High St, Kidlington, OX5 2DN, UK; 2 Brixton Health, Llwyngwril, Gwynedd, Wales, UK; 3 Epidemiology, Biostatistics and Clinical Research Centre, School of Public Health, Université libre de Bruxelles; 4 Centre for Tropical Medicine & Global Health, University of Oxford, UK; 5 KEMRI/Wellcome Trust Research Programme, Kilifi, Kenya; 6 Department of International Health, Johns Hopkins Bloomberg School of Public Health, Baltimore, USA; 7 USAID/Bureau of Humanitarian Assistance, USA; 8 IRD, UMI Résiliences, Paris, France; 9 Institut Pasteur, Epidémiologie des Maladies Emergentes, Paris, France; 10 FERDI, Université d’Auvergne, Clermont-Ferrand, France; 11 MRC/Wits Rural Public Health and Health Transitions Research Unit, School of Public Health, Faculty of Health Sciences, University of the Witwatersrand, Johannesburg, South Africa; 12 Harvard T.H. Chan School of Public Health, Boston, MA, USA; 13 Epicentre, Paris, France; 14 Departments of Pediatrics, Epidemiology and Biostatistics, University of California, San Francisco, USA; 15 Department of Nutrition, University of California, Davis, USA; 16 Division of Epidemiology & Biostatistics, University of California, Berkeley, USA; 17 The F.I. Proctor Foundation, University of San Francisco, San Francisco, USA; 18 Action Against Hunger USA, New York, NY, USA; 19 Friedman School of Nutrition Science and Policy, Tufts University, Boston, MA, USA; 20 Center for Health Research and Development, Society for Applied Studies, New Delhi, India; 21 Center for Human Nutrition, Department of International Health, Johns Hopkins Bloomberg School of Public Health, Baltimore, USA; 22 Center for Child, Adolescent and Maternal Health Research, Faculty of Medicine and Medical Technology, Tampere University, Tampere, Finland; 23 Department of Nutrition, Exercise and Sports, University of Copenhagen, Fredericksberg, Denmark

**Keywords:** Wasting, Stunting, Underweight, Mid-upper arm circumference, Anthropometry, Mortality, Therapeutic feeding

## Abstract

**Objective:**

To understand which anthropometric diagnostic criteria best discriminate higher from lower risk of death in children and explore programme implications.

**Design:**

A multiple cohort individual data meta-analysis of mortality risk (within 6 months of measurement) by anthropometric case definitions. Sensitivity, specificity, informedness and inclusivity in predicting mortality, face validity and compatibility with current standards and practice were assessed and operational consequences were modelled.

**Setting:**

Community-based cohort studies in twelve low-income countries between 1977 and 2013 in settings where treatment of wasting was not widespread.

**Participants:**

Children aged 6 to 59 months.

**Results:**

Of the twelve anthropometric case definitions examined, four (weight-for-age *Z*-score (WAZ) <−2), (mid-upper arm circumference (MUAC) <125 mm), (MUAC < 115 mm or WAZ < −3) and (WAZ < −3) had the highest informedness in predicting mortality. A combined case definition (MUAC < 115 mm or WAZ < −3) was better at predicting deaths associated with weight-for-height *Z*-score <−3 and concurrent wasting and stunting (WaSt) than the single WAZ < −3 case definition. After the assessment of all criteria, the combined case definition performed best. The simulated workload for programmes admitting based on MUAC < 115 mm or WAZ < −3, when adjusted with a proxy for required intensity and/or duration of treatment, was 1·87 times larger than programmes admitting on MUAC < 115 mm alone.

**Conclusions:**

A combined case definition detects nearly all deaths associated with severe anthropometric deficits suggesting that therapeutic feeding programmes may achieve higher impact (prevent mortality and improve coverage) by using it. There remain operational questions to examine further before wide-scale adoption can be recommended.

Undernutrition is a condition in which deficiency, or imbalance of energy, protein or other nutrients adversely affects body function and/or clinical outcome. Wasting and stunting are two manifestations of childhood undernutrition that remain common worldwide. The latest UN figures for 2021 estimate there are 47 million wasted children (6·9 million severely wasted) and 144 million stunted children at any one time^(1)^. Wasting and stunting are implicated in the deaths of almost two million children annually and account for over 15 % of all disability-adjusted life years lost in young children^(2)^. These figures are likely underestimates because the number of wasted children estimated in a population from cross-sectional prevalence surveys may miss many incident cases of this acute condition^(3,4)^

Recent years have seen an increased recognition that there is not one single ‘gold standard’ anthropometric definition of undernutrition, with current case definitions all having pros and cons. Over the last 5 years in particular, there has been increased scrutiny in the published literature of the widespread separation between the conditions of wasting and stunting among children in programmes, policy and research^(5–7)^. Although a number of gaps in the evidence base remain^(8)^, there is a growing understanding of the relationship between these two manifestations of undernutrition. In particular, it has been shown that wasting and stunting are often present in the same individuals^(9)^, they share a number of common underlying and immediate causal factors^(10,11)^ and one may precede the other^(12,13)^ with some evidence suggesting that stunting may be a biological response to previous episodes of wasting^(14)^. Reports have also highlighted that being both wasted and stunted concurrently, even at moderate levels, can be associated with considerable excess mortality, similar to that associated with severe wasting^(15–17)^. This heightened mortality risk does not appear to be explained by the severity of the child’s wasting and stunting alone^(12,16,17)^. Furthermore, concurrent wasting and stunting (WaSt) (defined as being both wasted, weight-for-height *Z*-score (WHZ) <−2 sd below the WHO standard median, and stunted, height-for-age *Z*-score (HAZ) <−2 sd below the standard median, at the same time) may affect up to 8 % of national populations of children aged 6–59 months^(18)^ with an estimated global burden of 16 million cases^(19)^.

The high mortality risk associated with this dual anthropometric deficit raises the question of whether children with WaSt should be included in therapeutic feeding service protocols if they are not already being reached. The findings also raise the question of how they may be identified in the community for admission into treatment. Historically, therapeutic services have treated children with severe acute malnutrition as currently defined by the WHO and UNICEF using three independent diagnostic criteria for children aged 6–59 months^(20)^: (1) mid-upper arm circumference (MUAC) <115 mm; or (2) WHZ <–3·0 or (3) the presence of bilateral pitting oedema.

A previous analysis, using data from a community-based cohort study assessing the risk of death in a representative sample of untreated children in Senegal in 1983–1984^(21)^, explored the diagnostic criteria needed to identify children at high near-term risk of death (i.e. within 6 months of measurement) including those concurrently wasted and stunted and therefore in need of treatment. Studies collecting data for such an analysis are no longer possible since the development of community-based management of acute malnutrition (CMAM) programmes capable of delivering effective severe acute malnutrition treatment at high levels of coverage have made it unethical to leave high-risk children untreated. The Senegal cohort mortality analysis found that two measures commonly used at community level, weight-for-age *Z*-score (WAZ) and MUAC, were independently associated with near-term mortality. Furthermore, the analysis found that if severely low MUAC (<115 mm) was used in combination with WAZ < −2·8, all deaths associated with severe wasting (WHZ < −3) and all deaths associated with WaSt could be predicted. It concluded that many of these deaths could likely be avoided by treatment in CMAM programmes and suggested that therapeutic treatment may achieve higher impact on averting mortality if a combination of WAZ and MUAC admission criteria was to be used in addition to the presence of nutritional oedema, rather than the current diagnostic admitting criteria. However, implications for increased caseloads were highlighted. The analysis also suggested that children identified by WAZ < −2·8 but with MUAC ≥ 115 mm may require lower-intensity treatment (i.e. lower dose and/or frequency of contact) than children identified using MUAC < 115 mm based on their demonstrated lower risk of near-term death.

Recognising that the results from a single cohort from one country may not be applicable more widely, this paper presents data from twelve similar mortality cohorts of largely untreated children including children suffering from wasting and WaSt. We present a multiple cohort individual-participant data meta-analysis of anthropometric criteria for the identification of children at elevated near-term mortality risk and explore the potential programmatic implications of applying the most appropriate criteria.

## Methods

### Data sources

The analysis presented here pools twelve cohort study datasets from twelve different low-income countries with data on children aged between 6 and 59 months collected between 1977 and 2013. The cohorts span countries in Africa, the Americas, Asia and the Pacific. Anthropometric measures were taken on children in the cohorts over periods ranging from 0 (when only a single anthropometric measurement was taken) to 37 months with a median of 12 months. All studies were large prospective cohort studies of children, or follow-ups of randomised trials examining various interventions and relationships including health and nutrition outcomes after vitamin A supplementation, health and nutrition outcomes after treatment with azithromycin for trachoma control and the distributions of mortality risk attributable to low nutritional status. All datasets were population-based samples of children selected based on age. In no case were the children selected based on any particular health or nutrition condition. Ten of these datasets were obtained by contacting the principal investigators of the studies featured in an earlier publication on single and multiple anthropometric deficits and mortality^(16)^. Permission to use the data was provided in all cases and access was given to original datasets (India and Nepal) or to datasets that were used in the earlier pooled analyses (all others). This dataset has been described in detail elsewhere^(22–29)^. Two additional datasets were obtained through personal contacts providing mortality cohort data from the Democratic Republic of Congo (DRC) and from Niger and are also described in detail elsewhere^(30–32)^. All data apart from that from Niger (2013) were collected in contexts and timeframes where widespread CMAM programming was not in place. All data were de-identified.

The characteristics of the included data in terms of location, study identifier, recruitment dates and age at recruitment as well as the numbers of children, follow-up periods and deaths within 6 months of measurement are shown in Table 1.

**Table 1:** Characteristics of the twelve cohort studies

Country	Study	Recruitment years	Children aged 6–59 months*	No. follow-up episodes†	Age (months) at recruitment median	Range	Age (months) at measurement median	Range	Duration (months) of anthropometric follow-up median	Range	Loss to follow-up over study duration (%)‡	No. deaths within 6 months of measurement§	MUAC collected?
Bangladesh	Arifeen (2001)	1993–1995	1317	3567	6	6–14	9	6–19	6	0–10	14	20	No
DRC	Van den Broek (1993)	1989–1993	4584	17 918	25	6–59	32	6–59	11	0–30	No info	196	Yes
Ghana	WHO/CHD (1998)	1995–1997	2615	5942	6	6–12	9	6–13	5	0–7	24·3	31	No
Guinea-Bissau	Molbak (1992)	1987–1990	985	4385	13	6–50	23	6–55	11	0–37	No info	118	No
India	WHO/CHD (1998)	1995–1996	3613	10 210	6	6–12	9	6–15	6	0–9	24·3	27	No
Indonesia	Katz (1989)	1977–1978	3806	17 367	27	6–59	33	6–59	15	0–18	7·8	214	No
Nepal	West (1991)	1989–1991	5883	25 159	21	6–59	33	6–59	16	0–25	6	128	Yes
Niger	O’Brien (2019)	2011–2013	970	1165	41	7–58	41	7–59	0	0–28	17·8	4	Yes
Peru	WHO/CHD (1998)	1995–1996	2289	5627	6	6–13	9	6–15	6	0–9	24·3	5	No
Philippines	Adair (1993)	1982–1983	2823	25 031	6	6–25	14	6–25	18	0–19	11·9	233	No
Senegal	Garenne (1987)	1983	5142	12 615	23	6–59	31	6–59	11	0–19	12·2	327	Yes
Sudan	Fawzi (1997)	1988	22 532	64 280	34	6–59	38	6–59	12	0–19	16	135	No
All	All	1977–2013	56 559	193 266	27	6–59	27	6–59	12	0–37	15·3‖	1438	4 of 12

MUAC, mid-upper arm circumference.

* Refers to the number of subjects used in the current analysis.

† Refers to the number of 6-month follow-up episodes used in the current analysis.

‡ For Ghana, India and Peru, the overall figure for three cohorts as reported in the original paper^(51)^ is given.

§ Refers to the number of events (deaths) used in the current analysis.

‖ Weighted average, with weight as the number of children age 6–59 months in each cohort.

Details on loss to follow-up were recorded in some, but not all, of the original cohorts. For the cohorts where reasons for loss to follow-up were recorded and reported on in the original study papers, outmigration was the most common reason given. See Table 1.

### Data management

Datasets were harmonised into a standard structure to contain the same variables with the same names, types, lengths, units and codes. Therapeutic programmes currently treat severe acute malnutrition primarily in children aged between 6 and 59 months with a standard protocol using ready-to-use therapeutic foods. Although community-based management of wasting in infants under 6 months of age is recommended by WHO, in practice, treatment is still often mainly inpatient based and active case finding is not common. The appropriate indicators to identify those most at risk of death and growth faltering and community-based management strategies are a focus of an active global community of policymakers, researchers and practitioners (MAMI Global Network) and hence merit separate analysis and discussion. The 6–59-month age group currently represents the vast majority of children treated in such programmes, therefore the analysis used only data for children in this age group at the time of anthropometric assessment. HAZ, WAZ and WHZ were calculated using the 2006 WHO growth standards^(33)^. Records with extreme *Z*-score values were identified and censored using the WHO ‘biological plausibility’ criteria^(34)^. Records with MUAC < 70 mm or > 240 mm were also censored on the grounds of biological implausibility. MUAC was measured and collected in four of the twelve cohorts (DRC, Nepal, Senegal and Niger). The outcome of interest was near-term mortality defined as death within 6 months of anthropometric assessment in line with previous similar analyses^(21)^. The units of analysis used are the 6-month period following an anthropometric measurement. Data management and analysis were performed using purpose-written R language (version 4.1.2) scripts managed using the R Analytic-Flow scientific workflow system (version 3.2.1) and including using the ‘zscorer’ function library in R. Data analysis scripts in the R language will be made available by the authors on request.

### Using anthropometry to predict mortality

Ten anthropometric case definitions were proposed by the members of the Emergency Nutrition Network’s Wasting and Stunting Technical Interest Group (WaSt TIG) for examination (see Table 2). With practical implications in mind, the initial rationale was to explore different ‘severe’ anthropometric deficits. This was then expanded to include moderate deficits, WHZ and MUAC, that are used for identification and admission into nutrition treatment programmes and HAZ and WAZ in response to peer reviewer request. Each of the case definitions was evaluated for its suitability for use as a case finding and admission criteria for therapeutic feeding programmes by investigating the ability of the case definition to predict mortality within 6 months of anthropometric assessment. This analysis was performed for all children and separately for children aged 6 to 23 months and children aged 24 to 59 months at anthropometric assessment. The units of analysis used in the analyses are the individual 6-month follow-up episodes.

**Table 2:** Pooled sensitivity, specificity and informedness (*Youden’s index)* for 12 anthropometric case definitions and death within 6 months of measurement in 12 cohorts

Age	Case-definition	Sensitivity (%)*	95 % CI†	Specificity (%)*	95 % CI†	Youden’s index (%)*	95 % CI†
6 to 23 months	HAZ < −3	27·92	18·18, 37·65	82·85	77·30, 88·41	11·75	6·17, 17·34
	HAZ < −2	52·94	42·47, 63·41	58·27	48·19, 68·36	12·30	6·46, 18·14
	WAZ < −3	30·25	22·11, 38·40	87·65	83·65, 91·65	18·58	11·87, 25·28
	WAZ < −2	56·14	46·86, 65·41	65·64	54·88, 76·39	22·50	15·56, 29·44
	WHZ < −3	11·91	7·16, 16·66	96·99	96·06, 97·91	8·78	4·54, 13·01
	WHZ < −2	30·48	20·64, 40·32	86·24	81·94, 90·54	16·93	9·75, 24·10
	MUAC < 115 mm‡	21·04	9·62, 32·46	92·10	87·07, 97·14	10·96	5·09, 16·83
	MUAC < 120 mm‡	31·81	13·50, 50·12	83·70	74·08, 93·31	13·79	4·18, 23·40
	MUAC < 125 mm‡	47·00	24·49, 69·52	70·99	56·14, 85·84	17·73	5·49, 29·97
	WHZ < −2 and HAZ < −2	20·54	8·46, 32·62	87·37	80·86, 93·88	9·37	2·55, 16·19
	MUAC < 115 mm or WHZ < −3‡	23·91	14·83, 32·98	89·50	85·58, 93·43	12·49	6·57, 18·40
	MUAC < 115 mm or WAZ < −3‡	37·91	24·37, 51·45	78·94	74·91, 82·98	18·55	7·99, 29·12
24 to 59 months§	HAZ < −3	48·41	35·50, 61·31	70·88	62·27, 79·49	22·51	18·09, 26·92
	HAZ < −2	73·82	61·97, 85·68	42·89	30·92, 54·85	19·32	13·29, 25·35
	WAZ < −3	31·10	25·52, 36·69	88·90	85·96, 91·85	22·26	18·03, 26·49
	WAZ < −2	60·07	52·28, 67·86	64·54	57·07, 72·01	28·09	22·70, 33·48
	WHZ < −3	7·83	4·22, 11·45	99·16	98·78, 99·53	6·64	3·50, 9·79
	WHZ < −2	22·74	14·48, 31·00	94·37	92·09, 96·64	17·32	11·78, 22·85
	MUAC < 115 mm	13·45	5·44, 21·47	98·49	97·19, 99·79	11·35	5·21, 17·50
	MUAC < 120 mm	18·71	7·81, 29·61	96·59	93·80, 99·38	14·24	7·47, 21·02
	MUAC < 125 mm	28·10	19·03, 37·17	92·47	86·59, 98·35	20·09	14·78, 25·40
	WHZ < −2 and HAZ < −2	19·68	13·65, 25·70	95·99	94·13, 97·86	16·12	11·36, 20·88
	MUAC < 115 mm or WHZ < −3	15·43	10·81, 20·06	97·80	96·31, 99·28	13·10	8·78, 17·43
	MUAC < 115 mm or WAZ < −3	32·81	26·98, 38·64	86·06	80·89, 91·22	23·69	18·04, 29·35
6 to 59 months	HAZ < −3	32·63	23·57, 41·69	79·30	72·30, 86·29	13·49	9·96, 17·02
	HAZ < −2	58·28	48·96, 67·60	54·26	43·75, 64·78	12·77	7·93, 17·60
	WAZ < −3	30·81	24·09, 37·52	88·62	85·07, 92·17	20·38	15·10, 25·66
	WAZ < −2	57·86	49·71, 66·00	66·28	56·91, 75·65	24·94	19·87, 30·02
	WHZ < −3	11·12	6·94, 15·30	97·96	97·43, 98·49	9·01	5·26, 12·76
	WHZ < −2	28·45	19·73, 37·16	90·06	87·34, 92·79	18·47	12·20, 24·75
	MUAC < 115 mm	17·56	7·60, 27·53	96·52	94·14, 98·90	13·46	6·49, 20·44
	MUAC < 120 mm	25·11	10·03, 40·18	92·64	87·72, 97·55	17·23	7·62, 26·83
	MUAC < 125 mm	37·22	20·30, 54·13	85·89	77·02, 94·76	23·00	13·77, 32·23
	WHZ < −2 and HAZ < −2	19·33	9·96, 28·70	93·55	90·25, 96·86	13·36	6·69, 20·02
	MUAC < 115 mm or WHZ < −3	20·38	13·58, 27·18	95·31	93·24, 97·38	15·00	10·12, 19·89
	MUAC < 115 mm or WAZ < −3	36·29	26·13, 46·45	83·56	79·10, 98·02	22·55	16·13, 28·97

HAZ, height-for-age *Z*-score; WAZ, weight-for-age *Z*-score; WHZ, weight-for-height *Z*-score; MUAC, mid-upper arm circumference.

* Cohort-specific results were combined using random effects (DerSimonian-Laird) meta-analysis.

† Intervals are expressed in ISO 31–11 form. The form [a;b] expresses the interval a ≤ x ≤ b. For example, (19·72, 37·97) is used to represent a 95 % CI that ranges between 19·72 and 37·97.

‡ Analysis restricted to cohorts that collected MUAC data (DRC, Nepal, Niger and Senegal).

§ Analysis restricted to cohorts that included data for ages ≥ 24 months (DRC, Guinea-Bissau, Indonesia, Nepal, Niger, Philippines, Senegal and Sudan).

Both quantitative and qualitative/semi-quantitative evaluation criteria were applied:

#### Sensitivity, specificity and informedness

The performance of different anthropometric indices and the twelve anthropometric case definitions for predicting death within 6 months of anthropometric assessment was evaluated by estimating sensitivity and specificity and plotting receiver operating characteristic curves. A measure of informedness (the probability of an informed decision being made as opposed to a random guess) combining both specificity and sensitivity in a single statistic (*Youden’s index* or *Youden’s J statistic*) was also calculated for each case definition^(35)^:

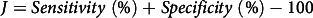




Cohort-specific results were combined using random effects (DerSimonion-Laird) meta-analysis. Analyses including the MUAC case definition (or where MUAC was a component of the case definition) were restricted to the four cohorts that collected MUAC data (DRC, Nepal, Niger and Senegal). Analysis for children over 24 months was restricted to the eight cohorts that included data for children aged ≥ 24 months (DRC, Guinea-Bissau, Indonesia, Nepal, Niger, Philippines, Senegal and Sudan).

#### High specificity

Case definitions with a point estimate of specificity below 80 % were rejected. The rationale for the use of this criterion for this analysis is to avoid programme caseloads being dominated by very large numbers of false-positive predictions when the outcome of interest is uncommon, as is the case with an outcome such as death within 6 months of anthropometric assessment.

#### Face validity

Children die for many reasons, but it is reasonable and common to try to help to prevent deaths in children with *severe* anthropometric deficits using therapeutic feeding. The standard used for severe anthropometric deficits was a *Z*-score < −3, MUAC below a threshold known to be associated with a mortality risk considerably above baseline (i.e. MUAC < 115 mm)^(20)^ or WaSt which is also known to be associated with a mortality risk considerably above baseline^(16,17,36)^.

#### Inclusivity

The possibility of identifying all, or nearly all, children with severe anthropometric deficits who are likely to die within 6 months of measurement was explored using a sets-based analysis. This used four-dimensional Venn diagrams with sets defined by the WAZ < −3, WHZ < −3, MUAC < 115 mm (when MUAC data was present) and the WaSt case definitions. Three-dimensional Venn diagrams (with WHZ < −3, WAZ < −3 and WaSt) were used when MUAC was absent from a cohort dataset. We included WHZ-based case definitions as WHZ < −3 is currently used in many national and international therapeutic feeding guidelines and in emergency settings by international non-governmental organisations. The ability of moderate case definitions (WAZ < −2 and MUAC < 125 mm) to identify children with severe anthropometric deficits who are likely to die was also explored for completeness/comparison.

#### Compatibility/practicability

This is the degree to which a case definition is compatible with current practices, tools and case-defining thresholds. Case definitions requiring the measurement of height might be considered to lack compatibility because the integrated management of childhood illnesses (IMCI) syllabus does not cover the measurement of height or the calculation/lookup of the WHZ or HAZ values, height boards are often not part of routine clinic equipment, and the measure requires at least two trained health staff who are often in short supply at community level.

Bilateral pitting oedema was not investigated because the relevant data was not present in the cohort datasets. It was assumed that therapeutic feeding programmes will continue to admit children using bilateral pitting oedema as an independent admission criterion due to its association with mortality and its response to treatment.

### Further analysis of mortality risk

The case definitions best meeting all the evaluation criteria (MUAC < 115 mm and WAZ < −3 – see results) were further examined by estimating and plotting mortality risks using risk ratios (RR) in groups of children meeting different components of that case definition:

- Children meeting only the MUAC component of the case definition (i.e. MUAC < 115 mm and WAZ ≥ −3)
- Children meeting only the WAZ component of the case definition (i.e. MUAC ≥ 115 mm and WAZ < −3)
- Children meeting both the MUAC and WAZ components of the case definition (i.e. MUAC < 115 mm and WAZ < −3)

The rationale for this analysis was that it is possible that within the proposed case definitions there may be subsets of children who are at lower risk of death and for whom a lower intensity and/or shorter duration of treatment may be appropriate. Risk ratios were calculated using 2 × 2 tables (exposure as case/non-case and outcome as died within 6 months/alive at or after 6 months) in each cohort and pooled using a random effects (DerSimonian-Laird) meta-analysis. Risk ratios for children within the proposed case definitions were plotted alongside that for children meeting the standard CMAM admission criteria of MUAC < 115 mm and MUAC < 125 mm for comparison. This analysis was also conducted by age category. This analysis might prove useful when designing interventions that use the selected case definition for trialling the possibility of lower intensity of treatment for some groups and exploring the effects that changing case definitions may have on programme caseloads and workload.

### Effects of changing case definitions on programme caseload and workload

The effects of changing case definitions on programme caseload and workload were investigated using a simple ‘what-if?’ simulation adapted from previously published methods^(21)^.

Two models used were:

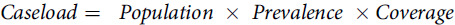




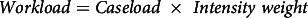

where:

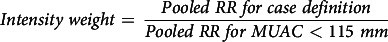




The intensity weight is a proxy measure of the expected required treatment intensity (i.e. contact frequency, quantity of therapeutic products, length of treatment) relative to current practice in CMAM programming (i.e. admitting on MUAC < 115 mm at community level) assuming treatment intensity depends upon mortality risk (i.e. children with higher mortality risk require more treatment and children with lower mortality risk require less treatment). Intensity weight used here is relative to the inputs required to treat children meeting the standard current CMAM admission criteria.

Prevalence is the proportion of the population meeting the case definition, and coverage is the estimated proportion of the eligible population reached by the relevant service (see below).

The population term was fixed at 17 000 assuming a service delivery unit (e.g. a health district) with a total population of 100 000 with 17 % of the total population aged between 6 and 59 months. The prevalence and coverage terms were modelled using fuzzy triangular numbers and fuzzy (i.e. interval) arithmetic to account for uncertainty in the point estimates. Ninety-five percent confidence intervals for the final results were calculated for a triangular distribution^(37)^.

Prevalence was modelled using the 25th, 50th and 75th percentiles of prevalence estimates for the MUAC and WAZ components of the selected case definition using data from 2 426 nutritional anthropometry surveys from fifty-one countries from 1992 to 2015^(17)^. Coverage for MUAC was modelled using the 25th, 50th and 75th percentiles of coverage estimates from 227 coverage assessments of CMAM programmes from twenty-nine countries from 2009 to 2017 recorded in a database provided by the Coverage Monitoring Network^(21)^.

It was assumed that cases with WAZ < −3 could be identified by growth monitoring or growth monitoring and promotion (GM/GMP) programmes with appropriate referral^(21)^. A literature review was undertaken and found useable coverage data for twenty-three GM/GMP programmes of differing scope and design from fourteen countries^(21)^. GM/GMP coverage was defined as a population coverage of more than three measurements in the previous 6 months in eligible children. It was assumed that coverage in lower socio-economic status groups (i.e. those groups most at risk of malnutrition) would be 80 % of the reported coverage. It was also assumed that the interface between GM/GMP programmes and CMAM programmes would be imperfect with 80 % of cases identified and referred by the GM/GMP programmes being admitted into CMAM programmes. Coverage of children with WAZ < −3 was modelled using the 25th, 50th and 75th percentiles of the resulting estimates.

The intensity weight term used to estimate workload was modelled using the observed pooled RR for each component of the selected case definition (i.e. MUAC < 115 mm, WAZ < −3) divided by the observed pooled RR for the standard CMAM admission criteria of MUAC < 115 mm used at community level. This assumes that children at lower risk of death will require lower intensity and/or lower duration of treatment. Uncertainty in the intensity weight term was modelled using fuzzy triangular numbers to cover the point estimate and 95 % confidence limits for the pooled RRs.

### Distributions of age, height-for-age Z-score and weight-for-height Z-score

The distributions of age, HAZ and WHZ in children meeting different components (MUAC and WAZ) of the proposed case definition were examined using boxplots.

## Results

### Using anthropometry to predict mortality

#### Sensitivity, specificity and informedness

Table 2 shows pooled sensitivity, specificity and informedness (*Youden’s Index*) for twelve anthropometric case definitions and all-cause mortality in twelve cohort datasets (four cohorts for MUAC). The analysis is split to show results by age group <24 months of age and ≥24 months (eight cohorts with data on children aged ≥24 months).

The table indicates that for children aged 6–59 months for all case definitions, specificity comes at the cost of sensitivity. Unsurprisingly, the most sensitive (i.e. identifying the most children who go on to die) measures, WAZ < −2 and HAZ < −2 are the least specific (misidentify the most children who are not going to die). However, four case definitions, (WAZ < −2), (MUAC < 125 mm), (MUAC < 115 mm **or** WAZ < −3) and (WAZ < −3) had the highest informedness (*Youden’s Index*) of all the tested case definitions. For the older age group (children 24–59 months), a fourth case definition (HAZ < −3) also has a similar level of informedness. For the younger age group, informedness is lower for most case definitions but the case definition (WAZ < −2) had the highest informedness.

**Fig. 1:**
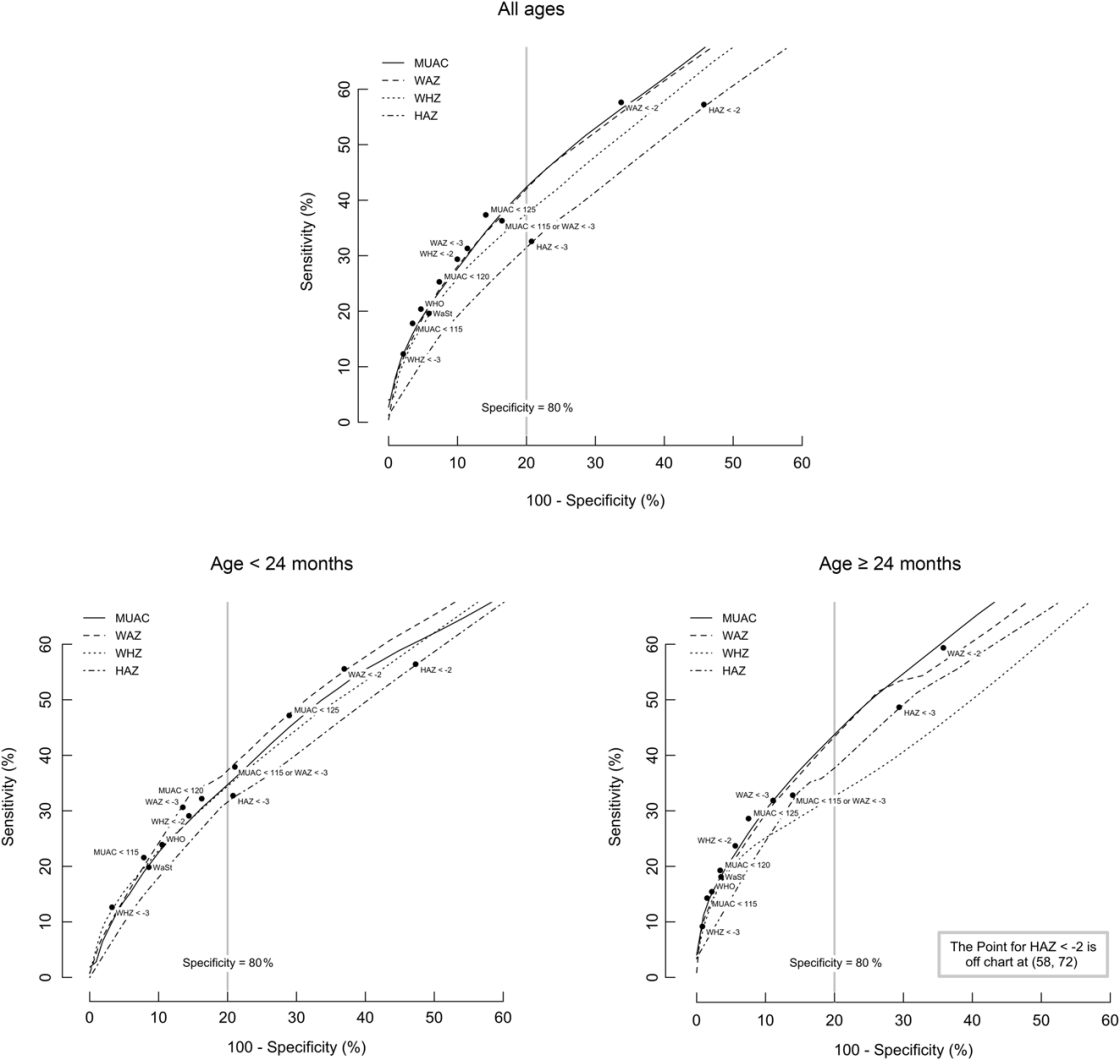
Pooled ROC curves for four anthropometric indices with sensitivity and specificity for twelve case definitions and death within 6 months of measurement in twelve cohorts and by age group. Note: Sensitivity and specificity were calculated for each measure in steps of 0·1 *Z*-scores (HAZ, WAZ, WHZ) or 1 mm (MUAC) and for each case definition (marked with ● and labelled) in each cohort and combined using a random effects (DerSimonian-Laird) meta-analysis. ‘WHO’ indicates MUAC < 115 mm or WHZ < −3 (current WHO admission guideline for CMAM programmes). WaSt indicates WHZ < −2 and HAZ < −2 (concurrent wasting and stunting). MUAC is measured in mm. The vertical solid line marks 80 % specificity. HAZ, height-for-age *Z*-score; WAZ, weight-for-age *Z*-score; WHZ, weight-for-height *Z*-score; MUAC, mid-upper arm circumference; CMAM, community-based management of acute malnutrition.

Figure 1 shows pooled receiver operating characteristic curves for four anthropometric measures with sensitivity and specificity for twelve case definitions and all-cause mortality in twelve cohorts (four cohorts for MUAC) and by age group. In all cases, MUAC and WAZ performed well, and the combined MUAC < 115 mm or WAZ < −3 case definition performed well in terms of both sensitivity and specificity for all children 6–59 months. For the <24 months age group, specificity for the MUAC < 115 mm or WAZ < −3 case definition was marginally, but not significantly, below the 80 % specificity standard outlined in our evaluation criteria.

#### Inclusivity

**Fig. 2:**
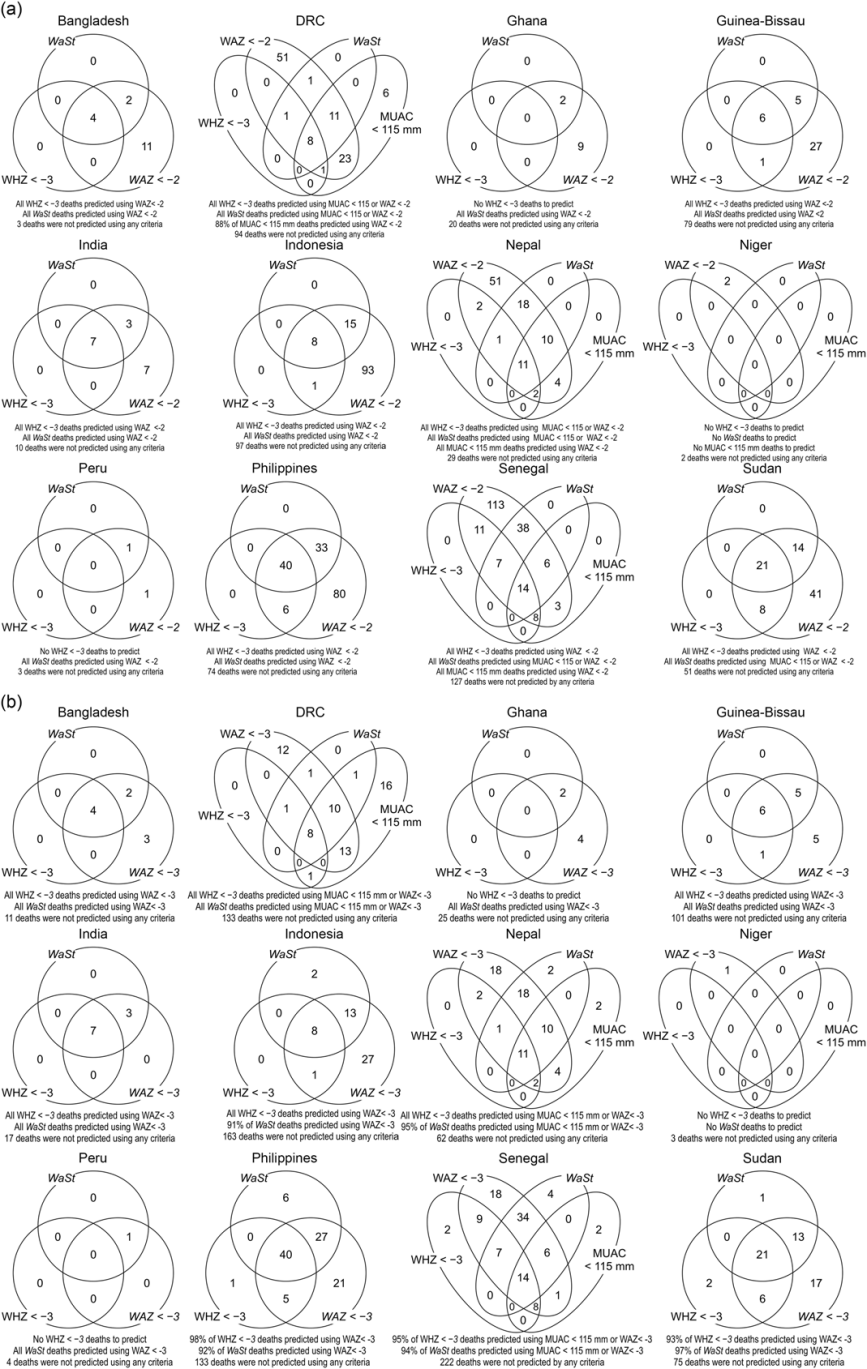
(a) Venn diagram analysis for the ability of MUAC < 115 mm (if present) **or** WAZ < −3 to predict deaths associated with WHZ < −3 or *WaSt* in twelve cohorts. (b) Venn diagram analysis for the ability of WAZ < −2 to predict deaths associated with WHZ < −3 or WaSt in twelve cohorts. Note: MUAC, mid-upper arm circumference; WAZ, weight-for-age *Z*-score; WHZ, weight-for-height *Z*-score.

**Fig. 3:**
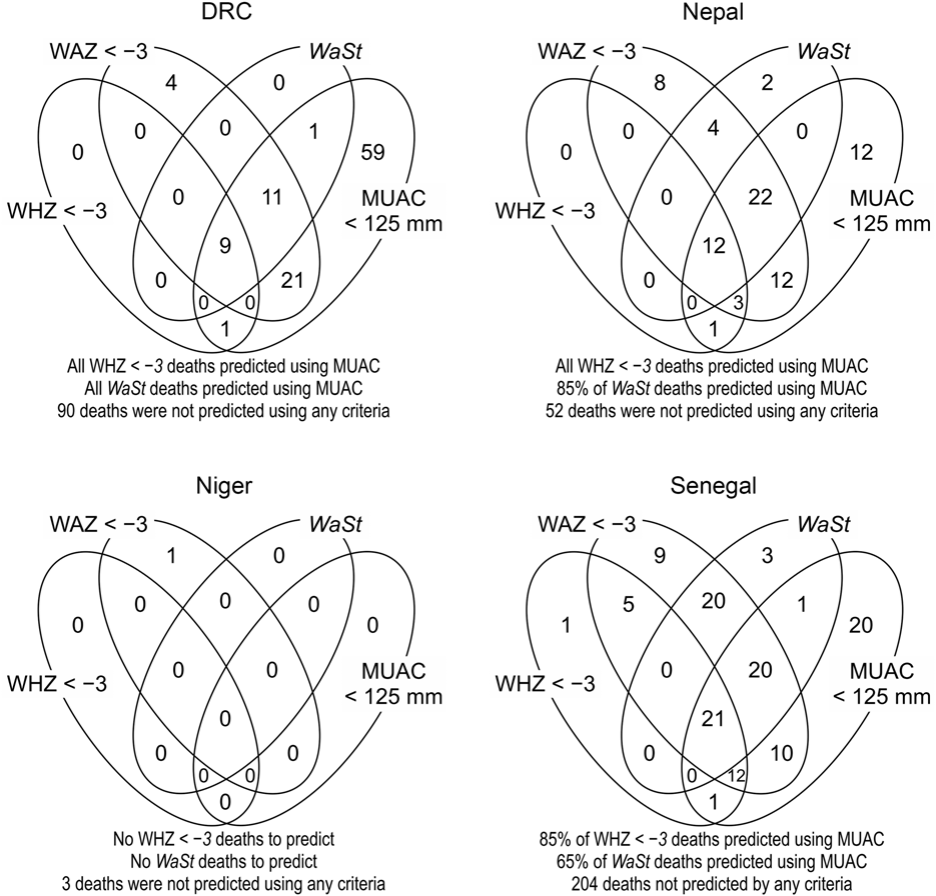
Venn diagram analysis showing that MUAC < 125 mm predicts all or nearly all deaths associated with WHZ < −3 and WaSt. MUAC, mid-upper arm circumference; WHZ, weight-for-height *Z*-score.

Figure 2a shows the results of the sets-based analysis of the ability of the case definition MUAC < 115 mm or WAZ < −3 to predict all, or nearly all, deaths associated with WHZ < −3 or WaSt. In interests of completeness, Fig. 2(b) shows similar analysis illustrating the ability of a moderate case definition WAZ < −2 to also predict all deaths associated with WHZ < −3 or WaSt, albeit with lower specificity. The use of WAZ < −2 was able to identify all deaths associated with severe anthropometric deficits, apart from 6 in the DRC cohort. Figure 3 shows the sets-based analysis of the ability of the MUAC < 125 mm case definition to predict all, or nearly all, deaths associated with WHZ < −3 or WaSt.

The ability of the combined case definition (i.e. MUAC < 115 mm or WAZ < −3) to predict deaths associated with WHZ < −3 or WaSt was only slightly better than that of the simpler WAZ < −3 case-definition. The use of MUAC < 115 mm did, however, predict additional deaths (i.e. 16 in DRC, two in Nepal and two in Senegal) that were not associated with WAZ < −3, WHZ < −3 or WaSt). In the sets-based analysis of MUAC < 125 mm (Fig. 3) conducted due to the increasing practice of implementing MUAC-only ‘simplified’ or ‘combined’^1^ protocols using this criterion, the MUAC < 125 mm criteria performed well at predicting all the WHZ < −3 deaths and almost all the WaSt deaths. The numbers shown in the cells of the Venn diagrams in Figs. 2 and 3 are counts of deaths.

Table 3 plots the above results alongside an assessment of the face validity and compatibility of each criteria/case definition. The case definitions, WAZ < −3 and MUAC <115 mm or WAZ < −3, both perform best according to the selection criteria used and were therefore the case definitions tested further.

**Table 3:** Summary of the evaluation of the suitability of different anthropometric case definitions for use as case-finding and admission criteria for therapeutic feeding programs

Criteria	Sensitivity	Specificity	Youden’s index	Face validity	Inclusivity	Compatibility (practicability)
HAZ < −3	●	○	○	●	?	○
HAZ < −2	●	○	○	○	?	○
WAZ < −3	●	●	●	●	●	●
WAZ < −2	●	○	●	○	●	●
WHZ < −3	○	●	○	●	○	○
WHZ < −2	○	●	●	○	○	○
MUAC < 115 mm	○	●	○	●	○	●
MUAC < 120 mm	○	●	●	?	?	○
MUAC < 125 mm	●	●	●	○	●	●
WHZ < −2 and HAZ < −2 (WaSt)	○	●	○	●	○	○
MUAC < 115 mm or WHZ < −3 (WHO CMAM criteria)	○	●	○	●	○	○
MUAC < 115 mm or WAZ < −3	●	●	●	●	●	●

● Sensitivity above median (i.e. ≥ 30·81 %); Specificity ≥ 80 %; Youden’s index above median (i.e. *J* ≥ 17·23 %) in the broadest (i.e. 6–59 months) age-group; others are based on qualitative assessment.

HAZ, height-for-age *Z*-score; WAZ, weight-for-age *Z*-score; WHZ, weight-for-height *Z*-score; MUAC, mid-upper arm circumference; CMAM, community-based management of acute malnutrition.

### Mortality risk for groups within the case definition mid-upper arm circumference < 115 mm or weight-for-age *Z*-score < −3

**Fig. 4:**
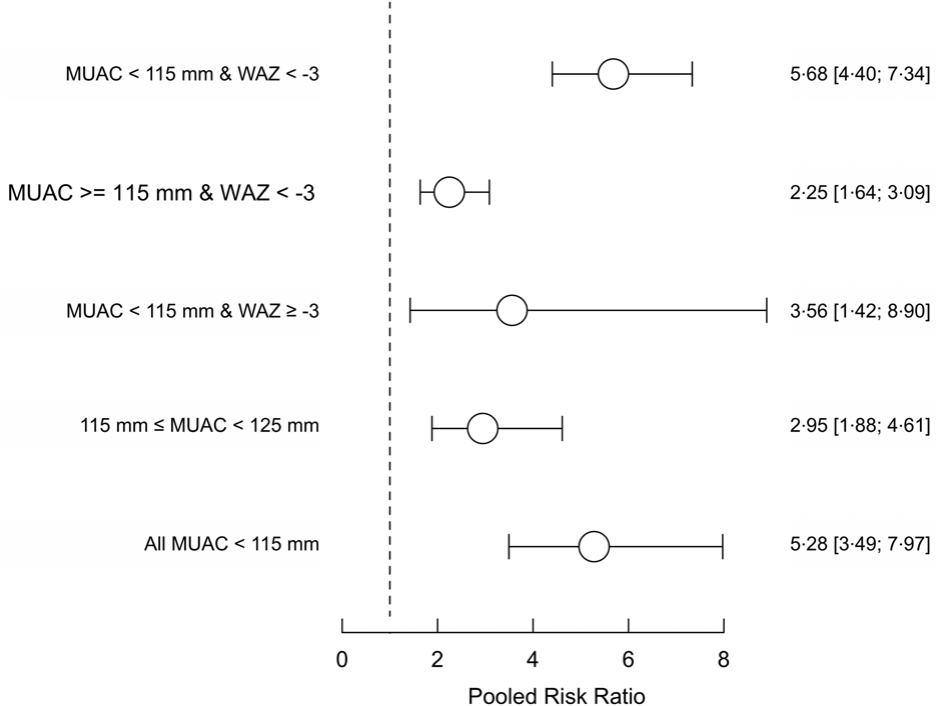
Pooled risk ratios of mortality for three mutually exclusive anthropometric case definitions (based on WAZ < −3 and/or MUAC < 115 mm) and death within 6 months of measurement in the four cohorts (i.e. DRC, Nepal, Niger and Senegal) that collected both MUAC and WAZ with existing MUAC criteria used in programming for comparison. Note: Point estimates and 95 % confidence limits are shown. The dashed vertical line marks the position of the null effect value (i.e. risk ratio = 1). WAZ, weight-for-age *Z*-score; MUAC, mid-upper arm circumference.

**Fig. 5:**
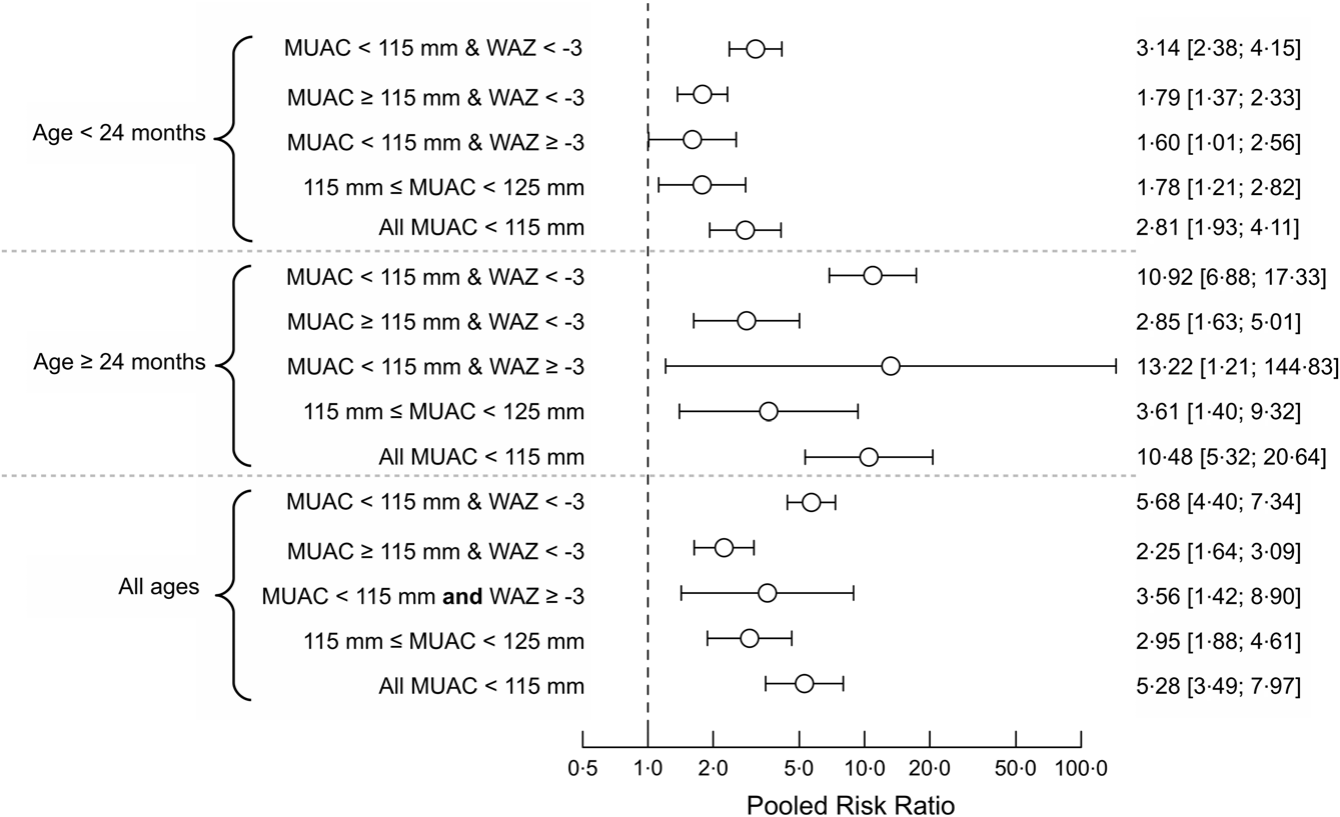
Pooled risk ratios by age group for three mutually exclusive anthropometric case definitions (based on WAZ < −3 and/or MUAC < 115 mm) and death within 6 months of measurement in three age classes in the four cohorts (i.e. DRC, Nepal, Niger and Senegal) that collected MUAC and WAZ. Note: Point estimates and 95 % confidence limits are shown. The dashed vertical line marks the position of the null effect value (i.e. risk ratio = 1). WAZ, weight-for-age *Z*-score; MUAC, mid-upper arm circumference.

Figure 4 shows the pooled relative risks for groups of children selected with the MUAC < 115 mm or WAZ < −3 case definition in the four cohorts (DRC, Nepal, Niger and Senegal) in which MUAC data was collected. Pooled relative risks for standard case definitions for therapeutic treatment using MUAC are included for comparison. The relative risks observed for the sets of children meeting only the MUAC component of the case definition or only the WAZ component of the case definition are lower than for the mortality risk observed for the standard therapeutic MUAC admission criteria. The relative risk in the set of children meeting both the MUAC and WAZ components of the case definition was similar to the mortality risk observed for the standard therapeutic MUAC admission criteria. Figure 5 shows the above analysis by age group. A similar pattern is seen in all age groups.

### Effects of changing case definitions on programme caseloads and workload

Table 4 shows the results of the simple ‘what-if?’ simulations of the effects of changing case definitions on programme caseload and workload.

**Table 4:** Results of the simple ‘what-if?’ simulations of the effects of changing case definitions on programme caseload and workload

	Case definition	Population	Prevalence (%)*	Coverage (%)†	Caseload*	Intensity weight*,†	Workload*,‡
A	All MUAC < 115 mm§	17 000	0·6, 1·3, 2·5	32·4, 41·6, 52·0	33, 92, 221	1·00	33, 92, 221
B	MUAC ≥ 115 mm & MUAC < 125 mm‖		3·4, 5·5, 8·8	32·4, 41·6, 52·0	187, 389, 778	0·24, 0·56, 1·32	45, 218, 1027
C	MUAC ≥ 115 mm and WAZ < −3¶		2·8, 5·0, 8·1	12·5, 21·8, 26·8	60, 185, 369	0·21, 0·43, 0·89	13, 80, 328

MUAC, mid-upper arm circumference; CMAM, community-based management of acute malnutrition; WAZ, weight-for-age Z-score.

* Parameter values and results are presented as fuzzy triangular numbers.

† Calculated by dividing the observed pooled risk ratio for each component of the selected case definition by the observed pooled risk ratio for the common CMAM case definition (A).

‡ Caseload adjusted (i.e. multiplied) by intensity weight.

§ This is a common admission criterion for CMAM programmes.

‖ Additional cases for combined (e.g. ComPAS) programmes above the common CMAM admission criteria of MUAC < 115 mm for children of all ages.

¶ Additional cases for programmes adding WAZ < −3 to the common CMAM admission criteria of MUAC < 115 mm for children of all ages.

The simulated caseload for programmes admitting on MUAC < 115 mm (i.e. A in Table 4) alone was 92 (95 % CI (50, 196)) cases per 17 000 population of children 6–59 months old. The simulated caseload for ‘combined’ programmes admitting on MUAC < 125 mm (i.e. A + B in Table 4) was 481 (95 % CI (291, 899)) cases per 17 000 population. The simulated caseload for programmes admitting on MUAC < 125 mm was therefore 5·23 (95 % CI (2·76, 25·99)) times larger than programmes admitting on MUAC < 115 mm. The simulated caseload for programmes admitting on MUAC < 115 mm or WAZ < −3 (i.e. A + C in Table 4) was 277 (95 % CI (141, 528)) cases per 17 000 population. The simulated caseload for programmes admitting on MUAC < 115 mm or WAZ < −3 was therefore 3·01 (95 % CI (1·48, 15·33)) times larger than programmes admitting on MUAC < 115 mm alone.

Caseload estimates do not account for varying intensities and durations of treatment that are likely to be required to treat children meeting different combinations of the components of the selected case definition. When caseloads were adjusted for mortality risk as a proxy for required intensity and/or duration of treatment, the simulated workload for programmes admitting on MUAC < 125 mm was 3·37 (95 % CI (2·03, 32·14)) times larger than programmes admitting on MUAC < 115 mm. The simulated workload for programmes admitting on MUAC < 115 mm or WAZ < −3 was 1·87 (95 % CI (1·03, 14·17)) times larger than programmes admitting on MUAC < 115 mm alone.

### Distributions of age, height-for-age *Z*-score and weight-for-height *Z*-score

**Fig. 6:**
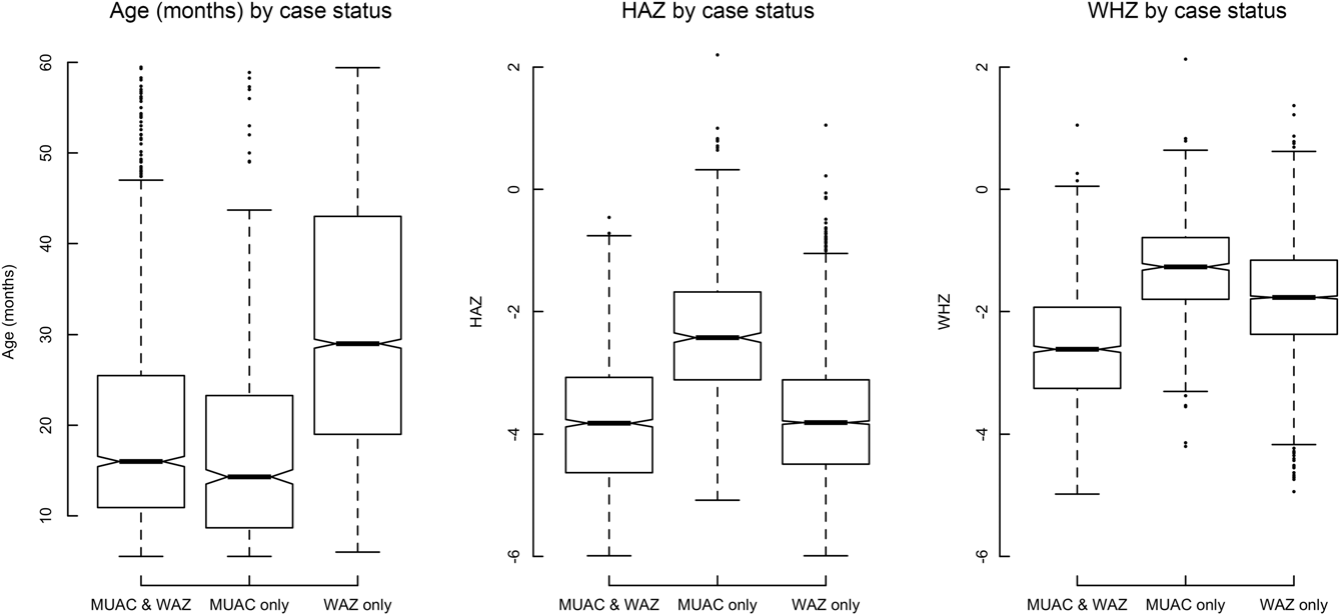
Distributions of age, HAZ and WHZ for three mutually exclusive anthropometric case-definitions (based on WAZ < −3 and/or MUAC < 115 mm) in children of all ages in the four cohorts (i.e. DRC, Nepal, Niger and Senegal) that collected both MUAC and WAZ. Note: For the above, the central boxes extend between the upper and lower quartiles with the thick line in the box marking the position of the median. The whiskers extend to 1·5 times the interquartile distance above and below the upper and lower quartiles and the isolated points mark the positions of data points more extreme than the range of values covered by the whiskers. The notches around the medians on each box show approximate 95 % confidence intervals; if they do not overlap then there is ‘strong evidence’ that two medians differ from each other. HAZ, height-for-age *Z*-score; WHZ, weight-for-height *Z*-score; MUAC, mid-upper arm circumference; WAZ, weight-for-age *Z*-score.

Figure 6 shows the distributions of age, HAZ and WHZ in children meeting the different combinations of the MUAC and WAZ components of the case definition examined using box plots. Children meeting the different combinations of the MUAC < 115 mm and/or WAZ <− 3 tended to differ from each other in terms of age, HAZ and WHZ.

## Discussion

The focus of this paper was the identification of the anthropometric deficits, measured at one point in time, that best predict mortality in the 6 months following measurement and therefore best guide the pragmatic identification of high-risk children in need of treatment. Of the twelve anthropometric case definitions evaluated above, two case definitions, WAZ < −3 and the combined case definition MUAC < 115 mm or WAZ < −3, met all the evaluation criteria. The latter case definition had the highest informedness (*Youden’s Index*) of case definitions meeting all the evaluation criteria and predicted all, or nearly all, deaths associated with WHZ < −3 or WaSt. This finding confirmed previous results^(21)^ and was replicated across all twelve cohorts of children that cover a range of contexts (Africa, the Americas, Asia and the Pacific) as well as different timeframes (1977 to 2013). While the focus of this analysis is on identifying severely malnourished children at the highest risk of mortality with a view to better addressing this, there are likely to be other positive outcomes of the effective and early identification and treatment for this group that are not examined here but are described elsewhere. These include reducing the risk of relapse and regression to wasting after treatment^(38,39)^, the prevention of further morbidity and poor cognitive development, potential impacts on linear growth^(40–42)^ and potential impacts on non-communicable diseases and overweight/obesity^(43,44)^.

WAZ is widely used in many countries across several community-based health and nutrition interventions including GMP as well as maternal child health and IMCI/IMNCI at primary health care facilities. The focus of humanitarian feeding programmes that have driven forward the adoption of community-based approaches to treating children over the last 20 years has been on identifying wasting only, therefore rejecting WAZ as an imperfect indicator for doing that. Our analysis suggests that a case definition that uses WAZ for the identification of severely malnourished children could use these established platforms to improve the identification and treatment of children at high risk of mortality. There are several well-known challenges with the age estimation part of the WAZ indicator in particular. These include increased difficulty of accurately estimating age as the child gets older leading to error and to ages being rounded to the nearest year, particularly in contexts where official documents such as birth certificates are not common and there are low-literacy rates. However, the use of a strengthened GMP platform, where infants are sought out early after birth and monitored regularly, rather than relying on individual measures taken in isolation, may help to minimise the impact of these challenges on over or under-diagnosis.

**Fig. 7:**
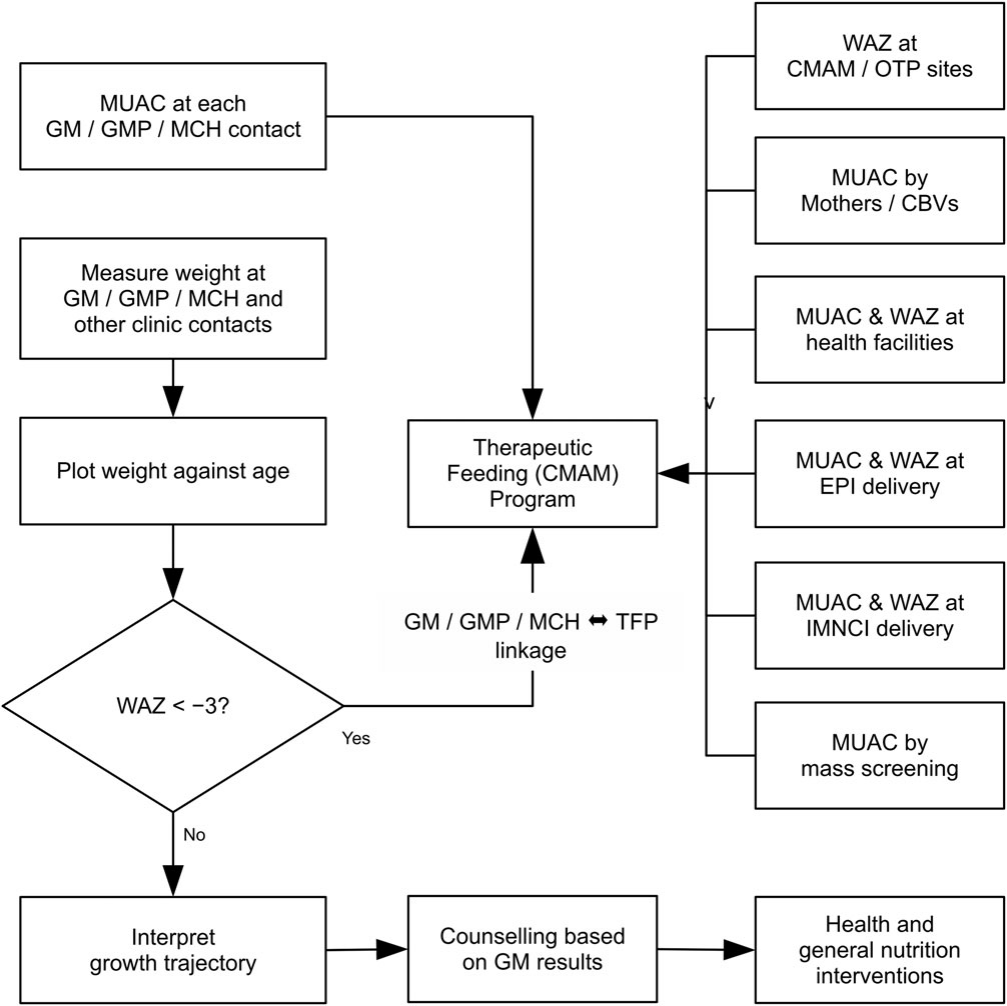
Potential programme model for linking Growth Monitoring and therapeutic feeding programmes and wider entry points in the health system, enabling the use of MUAC and WAZ as admission criteria^2^. Note: MUAC, mid-upper arm circumference; WAZ, weight-for-age *Z*-score.

The use of GMP as a platform to identify children at high risk of mortality may well lead to a selection bias towards younger children and infants who are better covered by the service. However, younger children are at greatest risk of death and other platforms illustrated in Fig. 7 can complement to ensure coverage across age groups. Such a model could also provide an optimised and integrated platform for the linking of preventative and treatment approaches for undernutrition in children. A vision for the programme platforms and referral mechanisms that could be used to operationalise any rollout of a model including this group of children in a treatment service is described in Fig. 7. This could be adapted according to programming realities in different contexts.

In addition, though our results indicate that the WHZ indicator is predictive of mortality in children, supporting concerns raised internationally about the move towards MUAC only programming for wasting treatment, the results of this analysis find that a combination of MUAC and WAZ was able to detect all near-term deaths associated with severe anthropometric deficits including WHZ < −3. Although WHZ is recommended by WHO for identification of severe wasting and thus included in many national protocols, the measurement of length/height is time-consuming, requires the presence of two skilled health workers and requires length boards/stadiometers which are often not available in many health facilities (as not included in IMCI) and therefore prohibitive for a service aiming to achieve decentralised coverage at low-level facilities. Furthermore, WHZ requires calculation of an index that has also been found to add an additional degree of complexity and increase error beyond the measurement error of the absolute measures, especially in younger children^(45)^. The findings of this paper suggest that the MUAC < 115 mm admission criterion in therapeutic feeding programmes should be retained and that consideration should be given to replacing the current facility-based WHZ admission criterion with a WAZ admission criterion that is able to identify those children at high risk of mortality and may be more compatible with existing community-based programming.

The different levels of mortality risk associated with the three groups of children selected by the combined case definition suggest that different levels of treatment intensity may be appropriate. This is important to consider given the probable increase in caseloads if the combined case definition were to be adopted by treatment programmes and the need to control programme costs and resource implications. Recent work by the ComPAS project group^(46)^ has found that children with a WAZ < −3 and a MUAC between 115 mm and 125 mm (i.e. children identified as having a relatively lower mortality risk than other groups meeting components of the combined case definition) do respond to treatment with over half achieving a WAZ > −3 and MUAC > 125 mm after 8 to 9 weeks of treatment on a reduced intensity protocol that provides a half dose ready-to-use therapeutic food ration (ready-to-use therapeutic food is a major cost-driver of CMAM programmes) and fortnightly contact. Recovery of WAZ and MUAC was achieved despite a higher level of stunting among these children (*v*. those admitted according to standard CMAM criterion). Appropriate discharge criteria and intensity of treatment for children admitted to therapeutic programmes using the combined case definition presented above needs to be further investigated.

Several other outstanding questions are highlighted by this analysis and indicate future work is needed to better understand the broader impacts of undernutrition on morbidity and mortality. We used a definition of near-term mortality risk as within 6 months of measurement; however, it is well known that timely identification is required to pick up incident cases of severe acute malnutrition and therefore the effect of frequency of measurement on the prognostic value of the case definitions examined in this paper for identifying mortality risk is the subject of further investigation. A number of papers have highlighted sex differences in anthropometric deficits and differences in WaSt in particular at population level, whereby boys are shown to be more vulnerable than girls^(12,17,40,18,47)^. Whether these population-level differences correspond to individual-level differences in the mortality risk associated with a given anthropometric deficit for boys compared to girls remains to be demonstrated. Our analysis indicated that for the younger age group, children age 6–23 months WAZ < −2 has high informedness. Though it scored lower than WAZ < −3 and the combined case definition across all judging criteria, it is possible that further investigation specific to the 6–23 months age group may be merited to see whether there is value in additional criteria for this age group. Finally, the question of the factors that are driving both wasting and stunting in these children at highest mortality risk is outstanding and remains the focus of other ongoing work^(48)^.

A particular strength of our study is that it is based on the analysis of a large number of available datasets from different contexts with anthropometric indices and mortality in community cohorts of untreated children. Due to the increased coverage of community-based management of acute malnutrition interventions globally over the last decade, more recent cohorts do not allow examination of the risk associated with anthropometric indicators in the absence of treatment. A large sample size is needed to study the relationship between anthropometric case definitions of severe undernutrition and the risk of death which are, from the statistical point of view, rare events. As such, this dataset is able to provide information regarding the prognostic value of anthropometric case definitions for identifying mortality risk in children across a range of populations. While this study does not include infants under 6 months, where the most predictive case definitions may be different, recent published work by others suggests that WAZ will be a better indicator than WHZ or WaSt for that age group^(49)^. As for older children, WAZ is easier to measure than weight for length in infants and, because the weight for length standards do not have *Z*-scores for infants <45 cm, WAZ will better capture mortality risk by including the most severely stunted neonates who may have low but incalculable weight for length ^(50)^.

There are also several limitations of the analysis presented in the paper. A potential bias may be introduced by loss to follow-up in the original studies masking deaths within the cohorts. Despite examination of the original papers (see Table 1), it was not possible to quantify this. It is also not possible to quantify the possible bias introduced by the absence of data on oedema which may affect the prognostic value of both WAZ and WHZ where it was present. It is also worth noting that the mortality results recorded are all-cause, as information on the cause of death was not consistently recorded between and within datasets. This means it is not possible to specifically attribute cause of death to anthropometric status. We can only ascertain whether anthropometric status is predictive of death. It is also possible that the monitoring of children in the cohorts and the potential referral for medical treatment may have led to lower mortality rates than in non-studied peers and may, therefore, underestimate relative risks. The caseload simulations presented are crude and results should be treated as broadly indicative of the magnitude and direction of the effect of changing case definitions on programme caseloads. In addition, the study included from Niger was carried out in a setting where children with MUAC < 115 mm were referred to treatment where they received nutritional support and may have received a single high dose of a broad-spectrum antibiotic with a long half-life effective against some bacterial infections including some (e.g. pneumonia) associated with considerable mortality. This may have improved the survival of these children as suggested by the small number of deaths seen in that study in children within 6 months of measurement. This may have led to an underestimation of the prognostic value of some of the case definitions. Similarly, two of the datasets (Sudan and Nepal) were from RCTs of vitamin A supplementation, and we did not adjust for the treatment group in the analysis of these datasets. In the Sudan trial, vitamin A supplementation did not have an effect on child growth or mortality; therefore, it is unlikely that this variable would influence the association between anthropometric deficits and mortality in a substantial way. However, in the Nepal trial, vitamin A significantly reduced the risk of mortality; thus, the exclusion of this variable from our analysis of this dataset may have led to RR estimates that underestimate the risk between anthropometric deficits and mortality. Finally, the generalisability of the results may be reduced based on the age of the studies, the majority of which were conducted >25 years ago. This is particularly so given broad secular trends in under-five mortality in the time since then which has been declining overall.

## Conclusion

The ability of the combined case definition (MUAC < 115 mm or WAZ < −3) to detect all, or nearly all, deaths associated with severe anthropometric deficits and the availability of programming platforms that already use these measures at community level suggests that therapeutic feeding programmes may achieve higher impact (i.e. prevent more mortality and improve the coverage of treatment for children at high risk of death) with this case definition. There remain, however, questions related to the intensity of treatment and operational implications in terms of increased caseloads/workload, costs, lengths of recovery times and feasibility of service delivery to balance with this potential for higher impact. These should be examined further before any wide-scale adoption of the case definition presented here.

## Footnotes

1
Whereby children meeting a MUAC ≥ 115 mm and MUAC <125 mm cut-off are treated through the same service as those with MUAC<115 mm but with lower intensity of treatment (less ready-to-use food, no antibiotic and less frequent contact).

2
GM, growth monitoring; GMP, growth monitoring and promotion; MUAC, mid upper arm circumference; WAZ, weight-for-age *Z*-score; EPI, expanded programme of immunisation, IMNCI, integrated management of newborn and childhood illness
